# Resveratrol ameliorates apoptosis induced by contrast medium ioxitalamate in HK-2 human renal proximal tubule cells in vitro

**DOI:** 10.1186/cc13573

**Published:** 2014-03-17

**Authors:** YY Chen, CC Cheng, TC Lin, Huang

**Affiliations:** 1 Buddhist Tzu Chi General Hospital, Hualien, Taiwan

## Introduction

Computed tomography with contrast medium is a common diagnostic tool in emergency and critical medicine. Contrast- induced nephropathy (CIN) is one of the leading causes of hospital- acquired acute kidney injury. Focus on renal tubule protection may be a hope to improve the effectiveness of current strategies.

## Methods

We used HK-2 human renal proximal tubule cells to evaluate the therapeutic potential of resveratrol, a polyphenol phytoalexin produced naturally by several plants, for contrast ioxitalamate-induced toxicity *in vitro. *Cytotoxicity was determined by MTT assay. Patterns of cell death were observed by flow cytometry. Cytoplasmic DNA fragmentation was examined by ELISA. Western blots were used to analyze the expression of related proteins.

## Results

**Figure 1:**
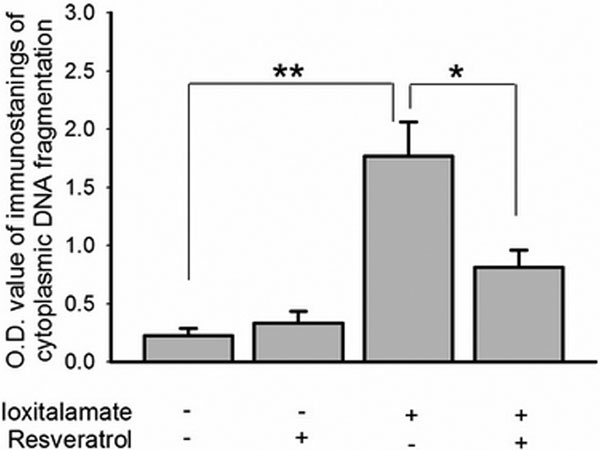
**Immunostaining of cytoplasmic DNA fragmentation in HK-2 cells**.

**Figure 2:**
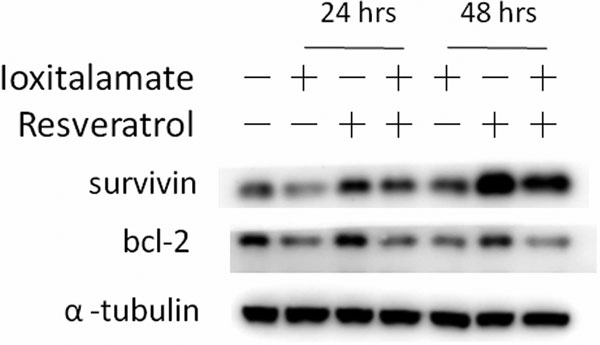
**Upregulated survivin expression by resveratrol**.

In a 48-hour administration, ioxitalamate elicited cytotoxicity on HK-2 cells. Annexin V^+ ^cells were significantly increased after 30 mg/ ml ioxitalamate exposure. A decrease in bcl-2 expression explained the ioxitalamate-induced apoptosis. Co-treatment with resveratrol ameliorated the cytotoxicity induced by ioxitalamate. Resveratrol at 12.5 μM decreased ioxitalamate-induced DNA fragmentation through upregulated expression of survivin. See Figure 1 and Figure 2.

## Conclusion

Resveratrol ameliorated apoptosis induced by contrast medium ioxitalamate in human renal proximal tubule cells. Investigations using animal models will be conducted in the future.

